# Association of volatile organic compounds with serum lactate dehydrogenase levels in the general adults

**DOI:** 10.1007/s00420-026-02215-5

**Published:** 2026-06-15

**Authors:** Ruitong Liao, Xiaoping Huang, Yue Xu, Qingya Zhao, Xiaogang Lv, Qi Liu, Qianqian Ji, Liuqing Li, Minghe Xu, Weiqing Chen, Yunzhang Wang, Shan Xu, Yiqiang Zhan

**Affiliations:** 1 Department of Epidemiology, School of Public Health (Shenzhen), Sun Yat-Sen University, Shenzhen, China; 2 Karolinska Institutet, Stockholm, Sweden; 3 Shenzhen Nanshan Center for Chronic Disease Control and Shenzhen, Nanshan Mental Health Center, Shenzhen, China; 4 Hangzhou Normal University, Hangzhou, China; 5 Guangdong Engineering Technology Research Center of Nutrition Transformation, Sun Yat-Sen University, Shenzhen, China

**Keywords:** Volatile organic compounds, Lactate dehydrogenase, Environmental exposure, Biomarkers of exposure

## Abstract

**Objectives:**

This study aimed to investigate the associations between urinary metabolites of Volatile organic compounds (mVOCs) and serum Lactate dehydrogenase (LDH) levels in a representative sample of the U.S. adults.

**Methods:**

We analyzed data from 4907 participants in NHANES 2011–2018. Seventeen urinary mVOCs were measured, and serum LDH was used as the outcome. Generalized linear models (GLM), restricted cubic splines (RCS), and weighted quantile sum (WQS) regression were used to assess linear, nonlinear, and mixture effects. Sensitivity analyses were conducted using urinary creatinine-corrected mVOC concentrations, and quantile g-computation (qgcomp) was applied as an alternative mixture modeling approach to validate the robustness of our findings. Furthermore, false discovery rate (FDR) correction was applied to account for multiple comparisons.

**Results:**

Several mVOCs were significantly associated with serum LDH. Notably, 2MHA, 34MHA, ATCA, and CYMA showed robust inverse associations, while PHEM was positively associated with LDH. RCS models revealed non-linear exposure–response relationships for multiple mVOCs. WQS regression indicated that both positively and negatively weighted mVOCs contributed to LDH levels, with DHBMA as a major positive contributor and 34MHA as a negative contributor. Most associations remained stable after sensitivity analyses.

**Conclusions:**

This study demonstrates that urinary mVOCs are associated with serum LDH levels, suggesting potential subclinical toxic effects of VOC exposure. These findings underscore the need for environmental risk management and further investigation into the biological pathways linking VOCs to systemic health outcomes.

**Supplementary Information:**

The online version contains supplementary material available at 10.1007/s00420-026-02215-5.

## Introduction

Volatile organic compounds (VOCs) are a broad class of carbon-containing chemicals characterized by high volatility at ambient temperatures (Irga et al. 2024). They are extensively produced and utilized in various domestic and industrial contexts (Atkinson and Arey 2003), including tobacco smoke, industrial emissions, vehicle exhaust, and volatile chemical products (McDonald et al. 2018), such as building materials and household products. Due to their widespread presence and potential toxicity, VOCs exposure has become a growing public health concern globally (Tsai 2019). Humans come in contact with VOCs via inhalation, ingestion, and dermal contact (Kampa and Castanas 2008). The cumulative effect of VOCs in the human body can cause various adverse health effects. Epidemiological and toxicological evidence has linked VOCs exposure to a variety of adverse health outcomes, including respiratory illnesses (Yoon et al. 2010; Ratiu et al. 2020; Paterson et al. 2021), cardiovascular diseases (McGraw et al. 2021; He et al. 2024; Kong and Qiu 2025), neurological diseases (Annavarapu and Kathi 2016; Werder et al. 2019), and autoimmune diseases (Ogbodo et al. 2022), etc. However, the underlying biological mechanisms through which VOCs exert systemic effects remain incompletely understood.

Lactate dehydrogenase (LDH) is a ubiquitous intracellular oxidoreductase that catalyzes the reversible conversion between pyruvate and lactate while regenerating NAD⁺ to sustain glycolysis under anaerobic conditions (Adeva-Andany et al. 2014). Clinically, LDH is widely regarded as a non-specific but sensitive biomarker of cell damage, tissue injury, and systemic toxicity (Wu et al. 2021; Xiao et al. 2023), and it holds significant diagnostic significance value in cancer and tumors (Claps et al. 2022; Jin et al. 2025). Elevated serum LDH reflects cellular injury or necrosis, as the enzyme is released into circulation following membrane disruption or metabolic stress (Drent et al. 1996).

Although the adverse health effects of VOCs exposure have been well recognized, its association with serum lactate dehydrogenase remains poorly understood. VOC exposure has been consistently shown to induce oxidative stress, mitochondrial dysfunction, and membrane lipid peroxidation—pathways that directly compromise cellular integrity and promote LDH leakage into the extracellular space.(Wu et al. 2025). Therefore, LDH serves as an integrative marker of early cytotoxicity and metabolic stress. While prior research has often focused on VOC-induced specific health outcomes or organ-specific biomarkers, such as alanine aminotransferase (ALT) for hepatotoxicity(Liu et al. 2023) and C-reactive protein (CRP) for inflammation (Zhang et al. 2024b), these endpoints typically reflect damage to a single organ system. In contrast, LDH offers a distinct advantage: it is a ubiquitous enzyme present in almost all body tissues, and its elevation in serum serves as a broad-spectrum indicator of cellular damage and metabolic disturbance (Farhana and Lappin 2023). Given that VOC exposure occurs through multiple routes and may affect various organ systems simultaneously (Shen et al. 2024), a systemic and non-organ-specific biomarker may be more appropriate for capturing early subclinical toxicity at the population level.

One previous study using canonical correlation with passive exposure monitors reported positive associations between certain ambient VOCs and LDH among other liver biomarkers (Liu et al. 2009). Studies on other environmental pollutants, such as particulate matter (Bai et al. 2019), heavy metals(Malik et al. 2021), and pesticide residue (Jaiswal et al. 2018), showed positive associations with serum LDH levels. Nevertheless, most existing studies have been limited to in vitro or small-scale experimental models, and large-scale population-based evidence remains scarce. The selection of LDH in this study addresses this gap by capturing the early, aggregate biological effects of mixed VOC exposure at the population level.

To address this knowledge gap, we conducted a comprehensive cross-sectional study using data from the U.S. National Health and Nutrition Examination Survey (NHANES) 2011–2018 to investigate the associations between urinary metabolites of volatile organic compounds (mVOCs) and serum LDH levels. We aim to clarify whether VOC exposure contributes to alterations in serum LDH at the population level and to evaluate LDH as a potential biomarker of subclinical systemic toxicity. In addition, we sought to identify the specific mVOCs that most strongly contributed to these associations. Our findings may provide new insights into the systemic health effects of VOCs and support risk assessment and public health strategies aimed at reducing exposure to harmful environmental chemicals.

## Methods

### Study design and population

The National Health and Nutrition Examination Survey (NHANES) is a rigorously designed cross-sectional study that draws a nationally representative sample of the United States population to evaluate health and nutritional conditions. All survey protocols were approved by the NCHS Research Ethics Review Board (CDC 2024). Detailed methodologies and protocols are publicly accessible through the official NHANES website (https://www.cdc.gov/nchs/nhanes/).

In this study, we merged data from four consecutive cycles of NHANES conducted between 2011 and 2018. A total of 11,564 participants had available urinary mVOC measurements, and serum LDH concentrations were measured in 26,673 individuals. Among them, 8883 participants completed both assessments.

**Fig. 1:**
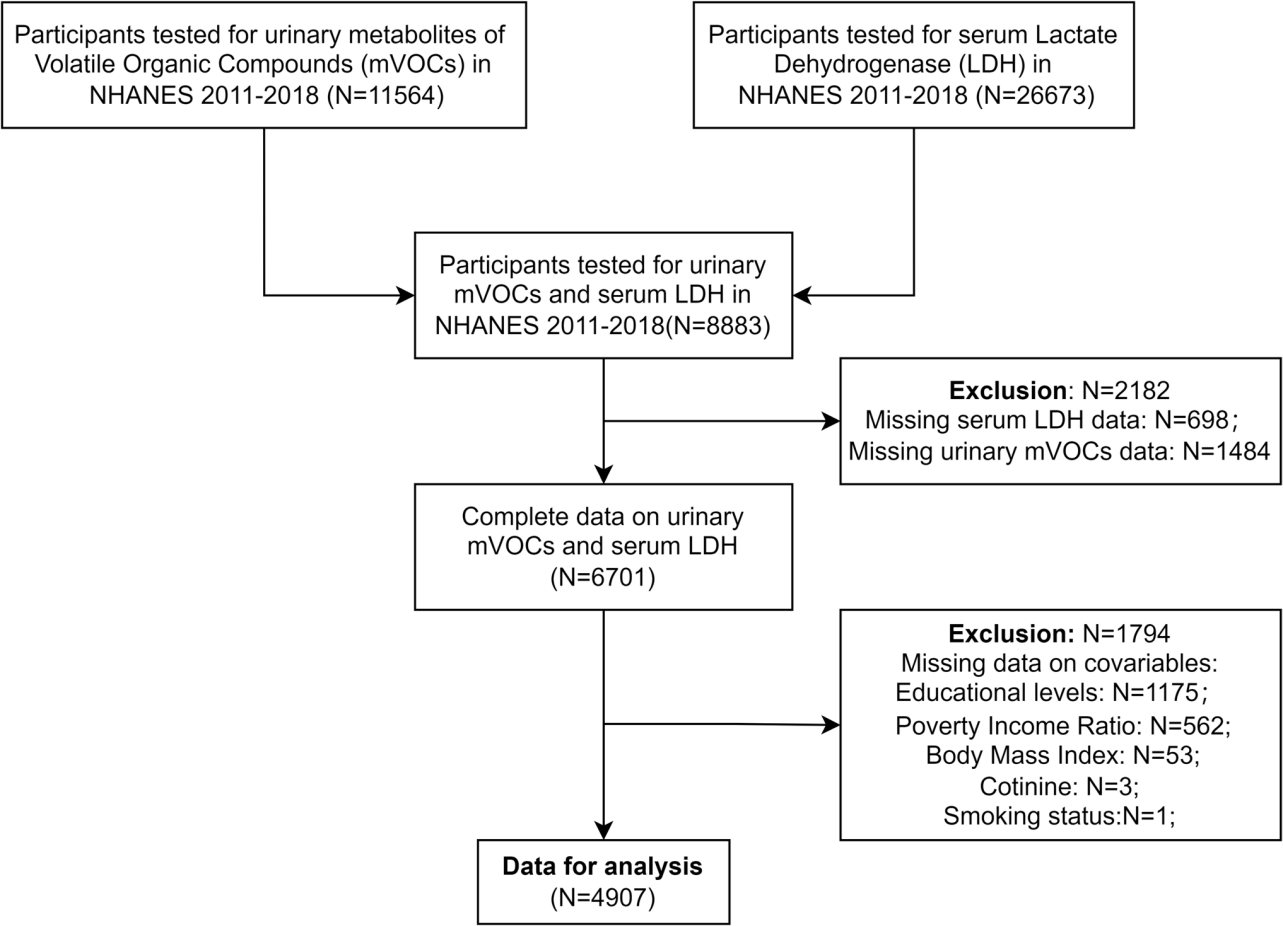
Flowchart of the study population from NHANES 2011–2018

Participants with incomplete or missing values for primary study variables were excluded to ensure the quality of the dataset. Specifically, 698 participants were excluded for missing LDH measurements, and 1484 were excluded due to missing data on one or more of the 17 targeted urinary mVOCs. An additional 1794 individuals were excluded due to incomplete covariate information. After removing individuals with incomplete information on key study variables, a total of 4907 participants were included in the final analysis (Fig. 1). All individuals included in the final analysis were between 20 and 80 years of age and had complete sampling weight information.

### Assessment of covariates

To minimize confounding, we adjusted an extensive array of demographic, socioeconomic, and health-related covariates into the models. The covariates comprised age, sex, race, education, cotinine, smoking and drinking status, family income-to-poverty ratio (PIR), body mass index (BMI), and hypertension or diabetes. These variables were measured using in-home interviews, structured questionnaires, and clinical evaluations performed at the Mobile Examination Center (MEC).

Race was grouped into Mexican American, other Hispanic, non-Hispanic White, non-Hispanic Black, or other. Educational attainment was divided into below high school, completed high school, and above high school. Smoking status was categorized as current, former, or never smoker. Drinking status was defined as heavy drinking or non-heavy drinking, with heavy drinking defined as consuming more than one alcoholic drink per day for women or more than two drinks per day for men, on average (Zhang et al. 2021). The PIR was categorized into three categories based on the federal poverty level: < 1.0, 1.0–3.5, and > 3.5. BMI was calculated by dividing weight by the square of height and classified according to the World Health Organization (WHO) classification (Consultation 2000): <  25.0 kg/m^2^, 25.0–29.9 kg/m^2^, and ≥ 30.0 kg/m^2^.

Furthermore, Diabetes was defined as fasting plasma glucose ≥ 6.99 mmol/L, use of insulin, or a prior physician diagnosis. Hypertension was identified as systolic blood pressure ≥ 140 mmHg, diastolic blood pressure ≥ 90 mmHg, a previous diagnosis, or current use of antihypertensive medication.

### Urinary mVOCs measurements

Across the 2011–2018 NHANES cycles, urinary metabolites of volatile organic compounds (mVOCs) were measured using ultra-performance liquid chromatography combined with electrospray tandem mass spectrometry (UPLC-ESI/MS/MS) (Alwis et al. 2012). For analyte concentrations falling below the lower limit of detection (LLOD), values were imputed as LLOD divided by the square root of 2. Detailed methods and information can be obtained by searching the official NHANES website.

There were 27, 27, 29, and 22 kinds of urinary mVOCs detected in the four cycles of 2011–2012, 2013–2014, 2015–2016, and 2017–2018, respectively. Among them, 19 mVOCs were consistently detected across all four cycles. Two compounds, *N*-ac-S-(2-carbomoyl-2-hydxel)-L-cys (GAMA) and *N*-ace-S-(2-hydroxyethyl)-L-cys (HEMA), were excluded to maintain adequate statistical power because their detection rates were less than 60% (Zhou et al. 2024). The remaining 17 mVOCs were included in the present analysis:2-Methylhippuric acid (2MHA), 3- and 4-Methylhippuric acid (34MHA), *N*-Acetyl-S-(2-carbamoylethyl)-l-cysteine (AAMA), *N*-Acetyl-S-(*N*-methylcarbamoyl)-L-cysteine (AMCA), 2-Aminothiazoline-4-carboxylic acid (ATCA), *N*-Acetyl-S-(benzyl)-l-cysteine (BMA), *N*-Acetyl-S-(*n*-propyl)-l-cysteine (BPMA), *N*-Acetyl-S-(2-carboxyethyl)-l-cysteine (CEMA), *N*-Acetyl-S-(2-cyanoethyl)-l-cysteine (CYMA), *N*-Acetyl-S-(3,4-dihydroxybutyl)-l-cysteine (DHBMA), *N*-Acetyl-S-(2-hydroxypropyl)-l-cysteine (HPM2), *N*-Acetyl-S-(3-hydroxypropyl)-l-cysteine (HPMA), Mandelic acid (MADA), *N*-Acetyl-S-(4-hydroxy-2-butenyl)-l-cysteine (MHB3), *N*-Acetyl-S-(1-phenyl-2-hydroxyethyl)-l-cysteine (PHEM), Phenylglyoxylic acid (PHGA), and *N*-Acetyl-S-(3-hydroxypropyl-1-methyl)-l-cysteine (HPMM).

These metabolites represent common biomarkers of exposure to benzene derivatives, halogenated hydrocarbons, aromatic hydrocarbons, and other volatile organic compounds. Table S1 lists the full names, abbreviations, parent compounds, and LLOD values for each mVOC.

### Serum LDH measurements

Serum lactate dehydrogenase (LDH) concentrations were measured using a kinetic rate method based on enzymatic oxidation. Blood specimens were collected from participants during their visits to the NHANES Mobile Examination Center (MEC), processed following standardized protocols, and stored at controlled temperatures until analysis. Detailed analytical procedures can be accessed in the NHANES Laboratory Data documentation.

### Statistical analysis

Baseline characteristics were described as means ± standard deviations for continuous variables and as proportions for categorical variables. To reduce skewness and improve normality, both urinary mVOC concentrations and serum LDH levels were log₁₀-transformed before analysis. We used Spearman correlation analyses to evaluate the relationships among individual mVOCs, and a heat map was constructed to visualize potential co-exposure patterns.

We applied three complementary statistical approaches to comprehensively assess the associations between urinary mVOCs and serum LDH levels: single-pollutant generalized linear model (GLM), restricted cubic splines (RCS), and weighted quantile sum (WQS) regression model.

Generalized linear models (GLM) were used to evaluate the relationship between single log-transformed mVOC concentrations and log-transformed serum LDH levels. Model 1 was unadjusted; Model 2 adjusted for age, sex, and race; and Model 3 was fully adjusted for age, sex, race, educational level, cotinine, smoking status, drinking status, PIR, BMI, and histories of hypertension and diabetes. To assess potential non-linear exposure–response relationships, restricted cubic spline (RCS) regression models were further constructed. The number of knots in the RCS models was selected by minimizing the Akaike Information Criterion (AIC), with optimal models generally using three to five knots.

The weighted quantile sum (WQS) regression model is a mixture analysis method that estimates the combined effects of multiple environmental exposures on health outcomes (Carrico et al. 2015). In this study, we employed a two-index Weighted Quantile Sum (2i-WQS) regression model to assess both positively and negatively associated exposure indices, thereby addressing situations in which some mVOCs may increase, while others may decrease, LDH levels. The model was fitted using 100 bootstrap samples, 10 quantile bins (*q* = 10), and a 60%/40% split for training and validation sets, and all covariates were adjusted for in the WQS analysis. The WQS indices and corresponding component weights were estimated using the R package “gWQS”.

To assess the robustness of our findings, we conducted two sets of sensitivity analyses. First, to account for inter-individual differences in urine dilution, we performed sensitivity analyses using urinary creatinine (UCr) -adjusted mVOC concentrations (each mVOC concentration divided by urinary creatinine and then log10-transformed) to minimize potential bias(O’Brien et al. 2016). Second, we applied quantile g-computation (qgcomp) to validate the robustness of the findings from the WQS. The qgcomp estimates the overall joint effect of multiple correlated exposures and relaxes the directional homogeneity assumption that is inherent in WQS regression (Keil et al. 2020).

In accordance with NHANES analytic guidelines, analyses incorporated the 2 year subsample MEC weights (WTSA2YR) for urinary VOC measurements. Because data from four survey cycles (2011–2018) were combined, the 2 year weights were recalculated by dividing WTSA2YR by four to generate appropriate multi-cycle sampling weights. All statistical analyses were performed using R software (version 4.3.2). Statistical significance was defined as a two-sided *p*-value below 0.05. To account for multiple comparisons across the 17 mVOCs in the primary generalized linear models, false discovery rate (FDR) correction (Glickman et al. 2014) was applied using the Benjamini–Hochberg procedure. Due to the exploratory or descriptive nature, statistical significance after FDR correction was defined as *q* < 0.10.

## Results

### Baseline characteristics of the participants

The baseline characteristics of the 4907 participants from NHANES 2011–2018 cycles are summarized in Table 1, stratified by sex. Participants were on average 47.4 years old, and 48.6% were male. Non-Hispanic Whites accounted for 66.3% of the cohort, reflecting the demographic distribution of the survey. Distributions of demographic, socioeconomic, and health-related variables were generally comparable between males and females. Additional baseline characteristics stratified by quartiles of serum LDH concentrations are provided in Table S2. Participants with higher LDH levels tended to be older, had a higher prevalence of obesity and hypertension, and were more likely to have lower educational attainment. The proportion of non-Hispanic Black participants also increased with higher LDH quartiles.

**Table 1:** Baseline characteristics of participants stratified by sex in NHANES 2011–2018

Characteristic	Overall, N = 4907	Male, N = 2433 (48.6%)	Female, N = 2474 (51.4%)
Age (years)	47.4 (16.7)	46.9 (16.6)	47.9 (16.8)
Race			
Mexican American	601 (7.8)	292 (8.3)	309 (7.4)
Other hispanic	513 (6.3)	236 (6.3)	277 (6.3)
Non-hispanic white	1860 (66.3)	939 (67.2)	921 (65.5)
Non-hispanic black	1098 (10.9)	556 (9.9)	542 (11.9)
Other race	835 (8.7)	410 (8.3)	425 (9.0)
Education			
Above high school	2814 (64.5)	1318 (62.1)	1496 (66.8)
Below high school	1002 (13.0)	540 (14.1)	462 (12.0)
Completed high school	1091 (22.5)	575 (23.8)	516 (21.3)
PIR			
< 1.30	1565 (22.2)	745 (20.0)	820 (24.2)
1.30–3.50	1825 (34.8)	913 (34.9)	912 (34.7)
> 3.5	1517 (43.0)	775 (45.0)	742 (41.2)
BMI (kg/m^2)			
< 25.0	1451 (29.0)	699 (25.5)	752 (32.3)
25.0–29.9	1542 (31.9)	876 (36.4)	666 (27.8)
≥ 30	1914 (39.1)	858 (38.1)	1056 (40.0)
Cotinine (ng/ml)	55.3 (129.7)	71.3 (153.2)	40.2 (100.5)
Smoking status			
Current smoker	938 (18.2)	537 (19.3)	401 (17.2)
Former smoker	1172 (25.3)	747 (31.6)	425 (19.4)
Never smoker	2797 (56.5)	1149 (49.1)	1648 (63.4)
Drinking status			
Heavy drinking	2693 (61.7)	1205 (54.0)	1488 (69.0)
No heavy drinking	2214 (38.3)	1228 (46.0)	986 (31.0)
DM			
No	4090 (87.0)	1999 (86.2)	2,091 (87.7)
Yes	817 (13.0)	434 (13.8)	383 (12.3)
Hypertension			
No	3160 (68.5)	1531 (67.5)	1629 (69.5)
Yes	1747 (31.5)	902 (32.5)	845 (30.5)

Values are shown as mean (standard deviation) for continuous variables and n (%) for categorical variables

BMI, body mass index; DM, diabetes mellitus; PIR, poverty income ratio

### Correlation of urinary mVOCs

**Fig. 2:**
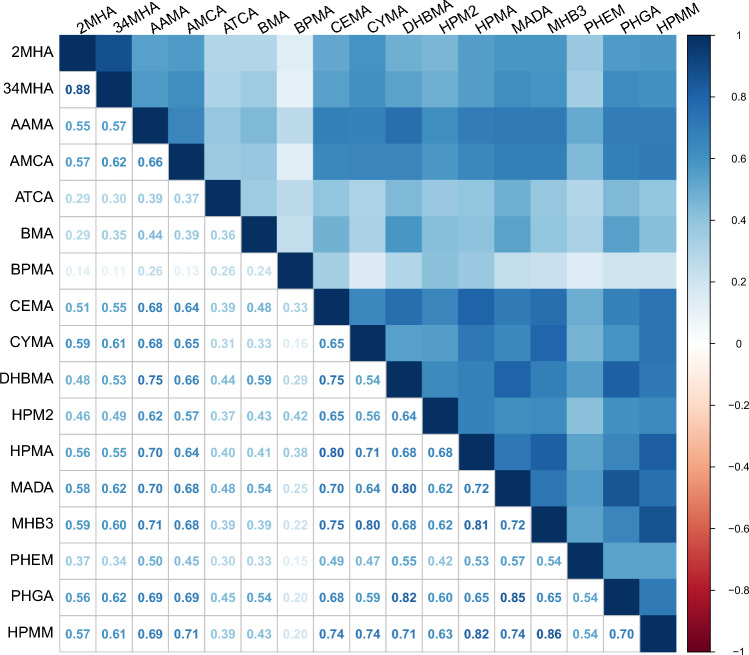
Spearman correlation coefficients among urinary VOC metabolites (mVOCs). The color intensity indicates the strength and direction of correlation, with blue representing positive and red representing negative correlations. All correlations were calculated based on log-transformed concentrations

Spearman correlation analysis revealed moderate to strong positive correlations among several mVOCs, especially those sharing common precursors or metabolic pathways (Fig. 2). For example, 2MHA and 34MHA, both metabolites of xylene, showed a strong correlation, as did HPMA and CEMA, metabolites of xylene. These findings indicate that co-exposure to multiple VOCs is common among the study population and support the use of mixture-based statistical approaches in subsequent analyses.

### Association of individual mVOCs with serum LDH tested by the GLM and RCS models

We applied generalized linear models (GLMs) to examine the associations between individual log₁₀-transformed urinary mVOCs and serum LDH levels, adjusting for progressively comprehensive sets of covariates in three models (Table 2). Based on the fully adjusted model (Model 3), we treated mVOCs as a categorical variable following the quartile distribution for analysis (Table S3). Restricted cubic spline (RCS) models were also applied to explore potential non-linear associations between mVOCs and LDH (Figs. S1, S2).

**Table 2:** Associations between urinary mVOCs and serum LDH levels in generalized linear models

Variable	Model 1		Model 2		Model 3	
	β (95% CI)	*P* value	β (95% CI)	*P* value	β (95% CI)	*P* value
2MHA	− 0.011 (− 0.016, − 0.006)	** < 0.001**	− 0.010 (− 0.015,− 0.005)	** < 0.001**	− 0.008 (− 0.013,− 0.002)	**0.005**
34MHA	− 0.017 (− 0.022,− 0.012)	** < 0.001**	− 0.018 (− 0.022,− 0.013)	** < 0.001**	− 0.018 (− 0.024,− 0.013)	** < 0.001**
AAMA	− 0.005 (− 0.011, 0.001)	0.121	− 0.001 (− 0.007, 0.005)	0.704	0.000 (− 0.006, 0.007)	0.952
AMCA	− 0.008 (− 0.014,− 0.002)	**0.009**	− 0.011 (− 0.016,− 0.005)	** < 0.001**	− 0.010 (− 0.017,− 0.004)	**0.002**
ATCA	− 0.015 (− 0.021,− 0.009)	** < 0.001**	− 0.014 (− 0.020,− 0.008)	** < 0.001**	− 0.015 (− 0.021,− 0.009)	** < 0.001**
BMA	0.007 (0.002, 0.013)	**0.012**	0.002 (− 0.003, 0.008)	0.419	0.001 (− 0.005, 0.007)	0.690
BPMA	− 0.007 (− 0.011,− 0.002)	**0.004**	− 0.004 (− 0.009, 0.000)	0.067	− 0.005 (− 0.010,− 0.001)	**0.017**
CEMA	0.004 (− 0.002, 0.010)	0.172	− 0.003 (− 0.010, 0.003)	0.284	− 0.004 (− 0.011, 0.002)	0.191
CYMA	− 0.008 (− 0.010,− 0.005)	** < 0.001**	− 0.006 (− 0.009,− 0.003)	** < 0.001**	− 0.007 (− 0.012,− 0.003)	**0.002**
DHBMA	0.017 (0.009, 0.025)	** < 0.001**	0.011 (0.003, 0.019)	**0.008**	0.007 (− 0.001, 0.015)	0.076
HPM2	− 0.008 (− 0.014,− 0.002)	**0.006**	− 0.007 (− 0.012,− 0.001)	**0.017**	− 0.005 (− 0.011, 0.001)	0.076
HPMA	− 0.005 (− 0.011, 0.000)	0.063	− 0.004 (− 0.010, 0.001)	0.125	− 0.002 (− 0.008, 0.005)	0.590
MADA	− 0.005 (− 0.012, 0.002)	0.175	− 0.005 (− 0.012, 0.002)	0.167	− 0.004 (− 0.011, 0.003)	0.245
MHB3	− 0.007 (− 0.012,− 0.002)	**0.010**	− 0.008 (− 0.013,− 0.003)	**0.001**	− 0.007 (− 0.014,− 0.001)	**0.027**
PHEM	0.020 (0.012, 0.029)	** < 0.001**	0.017 (0.009, 0.026)	** < 0.001**	0.021 (0.012, 0.030)	** < 0.001**
PHGA	0.006 (− 0.001, 0.013)	0.093	0.002 (− 0.005, 0.009)	0.533	0.002 (− 0.005, 0.009)	0.544
HPMM	− 0.006 (− 0.012,− 0.000)	**0.038**	− 0.009 (− 0.014,− 0.003)	**0.002**	− 0.007 (− 0.014,− 0.000)	**0.040**

Values are presented as β coefficients with 95% confidence intervals (CIs) and corresponding P values. Model 1: Crude model (unadjusted); Model 2: Adjusted for age, sex, and race; Model 3: Further adjusted for education level, Poverty Income Ratio (PIR), cotinine, smoking status, drinking status, Body Mass Index (BMI), and history of hypertension or diabetes

The values highlighted in bold indicate statistically significant differences (p < 0.05)

LDH, lactate dehydrogenase; mVOCs, urinary metabolites of volatile organic compounds

In Model 3, multiple mVOCs showed statistically significant associations with serum LDH levels. Specifically, 2MHA, 34MHA, AMCA, ATCA, CYMA, MHB3, and HPMM exhibited consistent and robust inverse associations, both as continuous variables and across quartiles. Among positively associated VOCs, PHEM, a metabolite of styrene, had the strongest and most consistent relationship with LDH (*β* = 0.021; 95% CI 0.012 to 0.030; *p* < 0.001), with a clear dose–response pattern across quartiles in Model 3 (*p* for trend < 0.001). Additionally, DHBMA was positively associated with LDH, although the effect was attenuated after full adjustment (Model 3: *β* = 0.007; *p* = 0.076).

Other metabolites, such as AAMA, CEMA, HPMA, MADA, PHGA, and HPM2, did not exhibit consistent or significant associations across all models.

RCS models further revealed several significant non-linear relationships, particularly for 2MHA, 34MHA, CYMA, DHBMA, HPMA, MHB3, and PHEM. For 34MHA and CYMA, U-shaped curves were observed, characterized by a decrease in LDH at low concentrations followed by an increase at higher exposure levels, suggesting potential threshold or compensatory effects. The p-values for overall and non-linear trends are summarized in Table S5. The full set of RCS curves for all 17 VOCs is provided in Figs. S1, S2.

Overall, our findings highlight that specific mVOCs, particularly those derived from exposure to toluene, xylene, acrylamide, and styrene/ethylbenzene, are significantly associated with serum LDH levels, supporting their potential role in systemic metabolic stress or inflammation.

### Association of urinary mVOCs with serum LDH tested on WQS regression

**Fig. 3:**
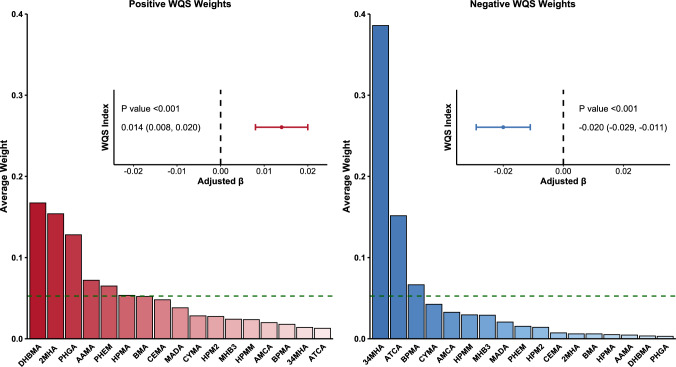
Weighted quantile sum (WQS) regression results showing the association between urinary VOC metabolite mixtures and serum LDH levels. Bar plots display the average WQS weights of individual urinary VOC metabolites contributing to the positive (left panel, red) and negative (right panel, blue) mixture indices. Dots and error bars represent the adjusted regression coefficients (β) and 95% confidence intervals of the WQS indices from fully adjusted models. Green dashed lines indicate the threshold for equal weight contribution (1 divided by the number of components)

After full adjustment, WQS regression revealed significant associations: the positive WQS index was significantly associated with higher serum LDH levels (*β* = 0.014; 95% CI 0.008 to 0.020), while the negative WQS index showed a significant inverse association (*β* =  − 0.020, 95% CI − 0.029 to − 0.011). These findings suggest that the combined exposure to VOCs exerts heterogeneous effects on LDH levels. The estimated mixture effects support the role of mVOCs as a composite environmental exposure influencing serum LDH levels. Contribution weights are shown in Fig. 3.

The positive WQS index, reflecting mVOCs positively associated with serum LDH, identified DHBMA as the top contributor by weight. Conversely, the negative WQS index, representing mVOCs inversely associated with LDH, was primarily driven by 34MHA. Notably, in fully adjusted linear models, DHBMA was positively but not significantly associated with LDH (*P* = 0.076). However, in WQS analysis, DHBMA received one of the highest positive weights, indicating its relative contribution to the overall mixture effect. This pattern suggests that DHBMA's effects may be amplified in combination with other VOCs or only manifest above a certain exposure threshold.

### Sensitivity analysis

Sensitivity analyses using UCr–corrected mVOCs [log10(mVOC/UCr)] yielded results largely consistent with the uncorrected models (Table S6, Figs. S3–S5). In addition, quantile g-computation (qgcomp) models confirmed the direction and magnitude of WQS findings (Fig. S6). Most associations persisted, including inverse associations for 34MHA, ATCA, and CYMA, and positive associations for PHEM. After applying the Benjamini–Hochberg false discovery rate correction across the 17 metabolites in the fully adjusted model (Table S7), the main associations remained robust.

## Discussion

To our knowledge, this is among the first large-scale, population-representative investigations to systematically examine the relationship between a comprehensive panel of urinary mVOCs and serum LDH levels, positioning LDH as a potential integrative biomarker of VOC-induced metabolic stress in the general population. While prior evidence linking VOCs to LDH has been largely confined to in vitro experiments or occupational cohorts with small sample sizes, our study integrates biomonitoring-based exposure assessment with systemic biochemical outcomes and mixture-oriented statistical modeling. By simultaneously evaluating individual metabolites, non-linear exposure–response patterns, and joint mixture effects, this study advances current understanding of VOC-related systemic toxicity beyond single-pollutant or organ-specific frameworks.

Overall, we observed significant associations between several urinary mVOCs and serum LDH concentrations, suggesting that internal VOC exposure may be linked to subclinical cytotoxic and inflammatory processes. Spearman correlation revealed co-exposure among VOCs, supporting mixture-based analyses. Consistently across GLM and quartile models, we found that 2MHA, 34MHA, CYMA, AMCA, and ATCA inversely associated with LDH, while PHEM showed positive associations. RCS models indicated non-linear patterns, with 34MHA and CYMA exhibiting U-shaped curves, suggesting threshold or adaptive effects. Mixture-based WQS models confirmed both positive and negative components, with DHBMA as a major positive contributor, and 34MHA as a negative contributor. Sensitivity analyses confirmed the robustness of most associations.

Our findings are in line with emerging evidence linking VOC exposure to systemic oxidative stress and cellular injury. For instance, PHEM’s consistent positive association with LDH is supported by experimental studies demonstrating styrene-induced cytotoxicity and LDH release in human broncho-alveolar cells (Mörbt et al. 2009). Similarly, DHBMA, a metabolite of 1,3-butadiene (BD), has been linked to increased oxidative DNA damage and subclinical cardiovascular stress in epidemiological studies (Lin et al. 2020; McGraw et al. 2021). Previous studies have also shown that BD exposure may trigger oxidative stress, leading to elevated levels of reactive oxygen species, and activate the mitochondrial apoptotic pathway (Yadavilli et al. 2007; Dong et al. 2024).

Other VOCs implicated in oxidative injury include xylene and acrylonitrile. Xylene is a widely used industrial solvent. A study shows that exposure to xylene can trigger oxidative stress, causing an increase in the levels of oxidative damage markers such as LDH, thereby leading to neuronal damage and behavioral defects (Han et al. 2025). Acrylonitrile, a pollutant that has been detected in drinking water, food products, and occupational environments, has been proven to cause cytotoxic effects and oxidative stress, and to release LDH (Mohamadin et al. 2005). However, the observed inverse associations for metabolites of xylene (2MHA, 34MHA), acrylonitrile (CYMA), cyanide (ATCA), and *N*, *N*-dimethylformamide (AMCA) are less well documented in the literature. First, one potential explanation may be a biphasic dose–response effect (Skaperda et al. 2022), where low-level exposure to VOCs triggers the upregulation of antioxidant defense mechanisms to prevent oxidative damage, thereby mitigating cellular damage and limiting LDH release. Specifically, low-dose redox stress may stimulate the nuclear factor erythroid 2–related factor 2 (Nrf2) signaling pathway (Calabrese and Kozumbo 2021). Nrf2 activation upregulates phase II detoxifying enzymes and antioxidant proteins, which collectively enhance the cellular capacity to neutralize reactive oxygen species (ROS) and maintain membrane integrity (Huang et al. 2015; Zhang et al. 2024a). This preconditioning effect could result in lower background levels of cellular damage and reduced LDH leakage at low VOC exposure levels, manifesting as an inverse association in epidemiological studies. Second, the observed inverse associations may reflect metabolic competition or pathway interference. Several VOCs share common metabolic pathways, particularly those involving cytochrome P450 enzymes such as CYP2E1 for bioactivation and glutathione conjugation for detoxification (Pohl and Scinicariello 2011). At low exposure levels, the presence of multiple VOCs may lead to competitive inhibition of metabolic activation, reducing the formation of toxic reactive intermediates and thereby decreasing cellular injury. Third, inter-individual variability in metabolic capacity may contribute to the observed inverse associations. Genetic polymorphisms in metabolic enzymes have been shown to significantly influence both the levels of urinary metabolites of VOC and the internal dose of reactive intermediates. For example, individuals with the GSTM1 null genotype exhibit higher urinary metabolite levels under low exposure conditions (Teixeira et al. 2004), while CYP2E1 variants are associated with reduced excretion of styrene metabolites (Carbonari et al. 2015). These differences in metabolic capacity could create inverse associations at the population level that reflect differential susceptibility rather than causal protective effects of VOC exposure. In contrast, high-level exposure to VOCs disrupts redox balance, resulting in toxicity via the emergence of oxidative stress and potentially in cell death. This phenomenon, hormesis, has been widely reported in toxicology (Lee and Jacobs 2015).

It is important to note that these inverse associations do not imply that VOC exposure is beneficial. Rather, they likely reflect the complex, non-linear dynamics of low-dose environmental exposures, where adaptive responses and metabolic competition produce patterns that diverge from the monotonic dose–response relationships observed in high-dose occupational or experimental settings. This interpretation aligns with our RCS findings of significant non-linearity for several metabolites and reinforces the notion that linear models may inadequately capture the full complexity of VOC-biology interactions.

Emerging toxicological and epidemiological evidence suggests that exposure to VOCs may influence LDH levels through several biological mechanisms. First, a substantial body of toxicological evidence suggests that VOCs can induce direct cytotoxicity in various cell types. In vitro studies have demonstrated that exposure to gasoline-related VOC mixtures can disrupt cell membrane integrity, resulting in significant LDH release from cells (Sayyed et al. 2022). Furthermore, a study has shown that the levels of LDH in the bodies of workers exposed to gasoline for a long time will significantly increase (Al-Faisal et al. 2010). Second, VOCs are known to stimulate oxidative stress, a major pathway of chemical-induced cellular injury (Yan et al. 2023). Upon inhalation or metabolic activation, many VOCs can generate reactive oxygen species (ROS), which damage lipids, proteins, and DNA (Dezest et al. 2017). Lipid peroxidation of cell membranes, in particular, compromises their structural integrity, facilitating LDH efflux into extracellular fluids. Third, some VOCs may elicit immune and inflammatory responses, involving the activation of transcription factors (Röder-Stolinski et al. 2008) and Pro-inflammatory genes, epigenetic modifications such as demethylation (Rehman et al. 2023), and upregulation of immune-regulatory molecules (Ogbodo et al. 2022).

Our study possesses several notable strengths. First, we assessed internal VOC exposure using urinary metabolites rather than relying on ambient measurements, thereby capturing biologically integrated exposure across multiple routes. Second, using a large, nationally representative sample from the NHANES database with rigorous quality control procedures, our findings possess high external validity and minimize potential selection bias. Third, our study leverages multiple statistical models, including GLMs, RCS, and WQS models to assess the combined effects of multi-VOC exposure and reflect real-world environmental exposures. The identification of U-shaped exposure–response patterns further contributes novel evidence regarding potential adaptive or threshold effects of low-dose VOC exposure. Finally, sensitivity analyses using UCr-corrected mVOC concentrations generally confirmed the main findings and enhanced the robustness of our exposure assessment. Collectively, these methodological and analytical strengths provide robust epidemiological evidence linking internal VOC burden to systemic biochemical alterations in the general population.

Nonetheless, several limitations warrant consideration. Firstly, the cross-sectional design limits the inference of causal relationships and cannot rule out reverse causality. Although our analyses were adjusted for numerous covariates, some degree of unaccounted or residual confounding remains. Moreover, there are inherent limitations in tracking the temporal trend of VOC exposure and serum LDH concentration. Because urinary mVOCs reflect short-term exposure windows, they may not accurately represent chronic or cumulative exposure, which could lead to exposure misclassification. Second, potential measurement errors in mVOCs quantification may arise from factors such as sample handling, storage duration, or analytical detection limits. Third, selection bias cannot be entirely excluded, as participants included in the VOCs subsample may differ from the overall NHANES population in ways related to health status or exposure profiles. Finally, although LDH is a useful marker of systemic damage, it is non-specific and may be influenced by other unmeasured physiological conditions. Future research should integrate longitudinal exposure assessment, multi-omics data, and toxicological experiments to elucidate the underlying mechanisms and strengthen causal interpretation.

## Conclusion

In summary, this study provides robust epidemiological evidence that exposure to certain VOCs, as reflected by their urinary metabolites, is significantly associated with serum LDH levels in U.S. adults. Both inverse and positive associations were identified, with non-linear patterns observed for several mVOCs. These results suggest that VOC exposure may induce subclinical tissue damage or systemic inflammation, as reflected by LDH elevation. Given the ubiquity of VOCs in the environment, our findings highlight the importance of continued monitoring and regulation of VOC exposure. From a broader perspective, these findings emphasize the necessity for improved monitoring systems, more rigorous emission controls, and tailored strategies to reduce human VOC exposure, particularly among susceptible populations. Further longitudinal and mechanistic studies are warranted to clarify causality and the biological pathways underlying VOC-related metabolic and inflammatory effects.

## Supplementary Information

Below is the link to the electronic supplementary material.

Supplementary Material 1.

## Data Availability

The data, code book, and analytic scripts used in this study are available upon request. All protocols and data are publicly accessible via the official NHANES website (https://www.cdc.gov/nchs/nhanes/).
