# Supplementary material for: Association of volatile organic compounds with serum lactate dehydrogenase levels in the general adults
Source: Int Arch Occup Environ Health. 2026 Jun 15;99(5):28. doi: 10.1007/s00420-026-02215-5 (PMC13269155; doi:10.1007/s00420-026-02215-5)

| **Table S1.** VOCs metabolites | | | | |
| --- | --- | --- | --- | --- |
| **Variable Name** | **Abbreviation** | **Parent Compound** | **VOCs Metabolite** | **LLOD (ng/mL)** |
| URX2MH | 2MHA | Xylene | 2-Methylhippuric acid | 5.00 |
| URX34M | 34MHA | Xylene | 3- and 4-Methylhippuric acid | 8.00 |
| URXAAM | AAMA | Acrylamide | N-Acetyl-S-(2-carbamoylethyl)-L-cysteine | 2.20 |
| URXAMC | AMCA | N, N-Dimethylformamide | N-Acetyl-S-(N-methylcarbamoyl)-L-cysteine | 6.26 |
| URXATC | ATCA | Cyanide | 2-Aminothiazoline-4-carboxylic acid | 15.0 |
| URXBMA | BMA | Toluene | N-Acetyl-S-(benzyl)-L-cysteine | 0.500 |
| URXBPM | BPMA | 1-Bromopropane | N-Acetyl-S-(n-propyl)-L-cysteine | 1.20 |
| URXCEM | CEMA | Acrolein | N-Acetyl-S-(2-carboxyethyl)-L-cysteine | 6.96 |
| URXCYM | CYMA | Acrylonitrile | N-Acetyl-S-(2-cyanoethyl)-L-cysteine | 0.500 |
| URXDHB | DHBMA | 1,3-Butadiene | N-Acetyl-S-(3,4-dihydroxybutyl)-L-cysteine | 5.25 |
| URXGAM | GAMA | Acrylamide | N-Acetyl-S-(2-carbamoyl-2-hydroxyethyl)-L-cysteine | 9.40 |
| URXHEM | HEMA | Ethylene oxide | N-Acetyl-S-(2-hydroxyethyl)-L-cysteine | 0.791 |
| URXHP2 | HPM2 | Propylene oxide | N-Acetyl-S-(2-hydroxypropyl)-L-cysteine | 5.30 |
| URXHPM | HPMA | Acrolein | N-Acetyl-S-(3-hydroxypropyl)-L-cysteine | 13.0 |
| URXMAD | MADA | Ethylbenzene, Styrene | Mandelic acid | 12.0 |
| URXMB3 | MHB3 | 1,3-Butadiene | N-Acetyl-S-(4-hydroxy-2-butenyl)-L-cysteine | 0.600 |
| URXPHE | PHEM | Styrene | N-Acetyl-S-(1-phenyl-2-hydroxyethyl)-L-cysteine N-Acetyl-S-(2-phenyl-2-hydroxyethyl)-L-cysteine | 0.700 |
| URXPHG | PHGA | Ethylbenzene, Styrene | Phenylglyoxylic acid | 12.0 |
| URXPMM | HPMM | Crotonaldehyde | N-Acetyl-S-(3-hydroxypropyl-1-methyl)-L-cysteine | 1.70 |
| mVOCs, metabolites of volatile organic compounds; LLOD, lower limit of detection | | | | |

| **Table S2.** Baseline characteristics of participants stratified by quartile of LDH in NHANES 2011-2018 | | | | | |
| --- | --- | --- | --- | --- | --- |
| **Characteristic** | **Overall**  N = 4907 | **LDH group** | | | |
|  |  | **Q1**, N = 1281 | **Q2,** N = 1173 | **Q3,** N = 1265 | **Q3,** N = 1265 |
| **Gender** |  |  |  |  |  |
| *Male* | 2,433 (48.6) | 616 (49.1) | 601 (49.9) | 615 (47.7) | 601 (47.7) |
| *Female* | 2,474 (51.4) | 665 (50.9) | 572 (50.1) | 650 (52.3) | 587 (52.3) |
| **Age (years)** | 47.4 (16.7) | 42.4 (15.5) | 45.8 (16.2) | 49.7 (17.0) | 52.4 (16.6) |
| **Race** |  |  |  |  |  |
| *Mexican American* | 601 (7.8) | 177 (8.0) | 142 (7.7) | 161 (8.3) | 121 (7.2) |
| *Other Hispanic* | 513 (6.3) | 147 (7.0) | 133 (6.2) | 131 (6.4) | 102 (5.3) |
| *Non-Hispanic White* | 1,860 (66.3) | 509 (67.3) | 461 (68.4) | 461 (66.3) | 429 (63.0) |
| *Non-Hispanic Black* | 1,098 (10.9) | 220 (8.9) | 241 (9.8) | 298 (11.1) | 339 (14.2) |
| *Other Race* | 835 (8.7) | 228 (8.8) | 196 (7.8) | 214 (7.9) | 197 (10.2) |
| **Education** |  |  |  |  |  |
| *Above High School* | 2,814 (64.5) | 811 (72.0) | 674 (65.1) | 677 (59.9) | 652 (60.2) |
| *Below High School* | 1,002 (13.0) | 226 (10.6) | 251 (14.2) | 285 (14.6) | 240 (12.9) |
| *Completed High School* | 1,091 (22.5) | 244 (17.4) | 248 (20.7) | 303 (25.5) | 296 (26.9) |
| **PIR** |  |  |  |  |  |
| *<1.30* | 1,565 (22.2) | 417 (23.1) | 374 (20.9) | 406 (22.4) | 368 (22.1) |
| *1.30-3.50* | 1,825 (34.8) | 445 (32.6) | 410 (31.2) | 473 (35.2) | 497 (40.7) |
| *>3.50* | 1,517 (43.0) | 419 (44.2) | 389 (47.9) | 386 (42.4) | 323 (37.2) |
| **BMI (kg/m^2)** |  |  |  |  |  |
| *<25.0* | 1,451 (29.0) | 457 (36.5) | 364 (30.2) | 378 (29.2) | 252 (18.7) |
| *25.0-29.9* | 1,542 (31.9) | 425 (33.4) | 370 (31.6) | 388 (30.6) | 359 (32.1) |
| *≥30* | 1,914 (39.1) | 399 (30.0) | 439 (38.2) | 499 (40.2) | 577 (49.2) |
| **Cotinine (ng/ml)** | 55.3 (129.7) | 58.1 (129.6) | 56.4 (129.0) | 50.5 (127.9) | 56.2 (132.8) |
| **Smoking status** |  |  |  |  |  |
| *Current Smoker* | 938 (18.2) | 287 (21.0) | 238 (18.4) | 211 (15.8) | 202 (17.5) |
| *Former Smoker* | 1,172 (25.3) | 267 (24.4) | 261 (22.2) | 320 (26.9) | 324 (28.0) |
| *Never Smoker* | 2,797 (56.5) | 727 (54.6) | 674 (59.5) | 734 (57.3) | 662 (54.4) |
| **Drinking status** |  |  |  |  |  |
| *Heavy drinking* | 2,693 (61.7) | 732 (63.5) | 673 (62.6) | 666 (59.5) | 622 (60.9) |
| *No heavy drinking* | 2,214 (38.3) | 549 (36.5) | 500 (37.4) | 599 (40.5) | 566 (39.1) |
| **DM** |  |  |  |  |  |
| *No* | 4,090 (87.0) | 1,095 (88.2) | 1,006 (88.7) | 1,046 (87.1) | 943 (83.6) |
| *Yes* | 817 (13.0) | 186 (11.8) | 167 (11.3) | 219 (12.9) | 245 (16.4) |
| **Hypertension** |  |  |  |  |  |
| *No* | 3,160 (68.5) | 984 (79.7) | 805 (72.7) | 778 (66.7) | 593 (53.1) |
| *Yes* | 1,747 (31.5) | 297 (20.3) | 368 (27.3) | 487 (33.3) | 595 (46.9) |
| Data are presented as mean ± SD or n %; | | | | | |
| LDH: Lactate Dehydrogenase; BMI: Body Mass Index; DM: Diabetes Mellitus; PIR: Poverty Income Ratio | | | | | |
|  | | | | | |

| **Table S3.** Association between urinary mVOCs and serum LDH by exposure quartiles in adjusted linear regression models | | | | | | | | | | | | | |
| --- | --- | --- | --- | --- | --- | --- | --- | --- | --- | --- | --- | --- | --- |
| **Variable** | **Q1** |  | **Q2** | |  | **Q3** | |  | **Q4** |  |  | ***P* for trend** |  |
|  |  |  | **β (95% CI)** | ***P* value** |  | **β (95% CI)** | ***P* value** |  | **β (95% CI)** | ***P* value** |  |  |  |
| **2MHA** | Ref |  | -0.001 (-0.008, 0.007) | 0.835 |  | -0.011 (-0.019, -0.004) | **0.004** |  | -0.016 (-0.023, -0.008) | **<0.001** |  | **<0.001** |  |
| **34MHA** | Ref |  | 0.002 (-0.006, 0.009) | 0.667 |  | -0.019 (-0.027, -0.012) | **<0.001** |  | -0.021 (-0.028, -0.013) | **<0.001** |  | **<0.001** |  |
| **AAMA** | Ref |  | 0.003 (-0.005, 0.010) | 0.489 |  | -0.000 (-0.008, 0.007) | 0.948 |  | -0.006 (-0.014, 0.002) | 0.123 |  | 0.965 |  |
| **AMCA** | Ref |  | -0.001 (-0.008, 0.007) | 0.871 |  | -0.006 (-0.014, 0.002) | 0.117 |  | -0.006 (-0.014, 0.002) | 0.117 |  | **0.023** |  |
| **ATCA** | Ref |  | -0.002 (-0.009, 0.006) | 0.665 |  | -0.017 (-0.024, -0.009) | **<0.001** |  | -0.017 (-0.025, -0.009) | **<0.001** |  | **<0.001** |  |
| **BMA** | Ref |  | -0.003 (-0.011, 0.004) | 0.394 |  | 0.002 (-0.006, 0.009) | 0.650 |  | 0.007 (-0.001, 0.015) | 0.071 |  | 0.905 |  |
| **BPMA** | Ref |  | -0.007 (-0.014, 0.001) | 0.090 |  | -0.005 (-0.012, 0.003) | 0.236 |  | -0.009 (-0.017, -0.002) | **0.015** |  | 0.088 |  |
| **CEMA** | Ref |  | -0.001 (-0.009, 0.006) | 0.714 |  | 0.004 (-0.004, 0.011) | 0.356 |  | 0.006 (-0.002, 0.014) | 0.117 |  | 0.789 |  |
| **CYMA** | Ref |  | -0.006 (-0.013, 0.002) | 0.140 |  | -0.009 (-0.016, -0.001) | **0.023** |  | -0.020 (-0.028, -0.013) | **<0.001** |  | **<0.001** |  |
| **DHBMA** | Ref |  | -0.000 (-0.008, 0.008) | 0.999 |  | 0.008 (0.000, 0.015) | **0.046** |  | 0.013 (0.005, 0.020) | **0.001** |  | 0.099 |  |
| **HPM2** | Ref |  | -0.004 (-0.011, 0.004) | 0.348 |  | -0.008 (-0.016, -0.001) | **0.032** |  | -0.011 (-0.018, -0.003) | **0.006** |  | **0.035** |  |
| **HPMA** | Ref |  | -0.000 (-0.008, 0.007) | 0.926 |  | -0.006 (-0.014, 0.001) | 0.097 |  | -0.007 (-0.015, 0.000) | 0.065 |  | 0.265 |  |
| **MADA** | Ref |  | -0.002 (-0.010, 0.006) | 0.593 |  | -0.000 (-0.008, 0.008) | 1.000 |  | -0.004 (-0.012, 0.003) | 0.261 |  | 0.568 |  |
| **MHB3** | Ref |  | 0.004 (-0.004, 0.012) | 0.301 |  | -0.003 (-0.011, 0.005) | 0.429 |  | -0.008 (-0.016, -0.001) | **0.036** |  | **0.012** |  |
| **PHEM** | Ref |  | 0.100 (0.093, 0.107) | **<0.001** |  | 0.012 (0.004, 0.019) | **0.002** |  | 0.029 (0.022, 0.035) | **<0.001** |  | **<0.001** |  |
| **PHGA** | Ref |  | 0.003 (-0.005, 0.010) | 0.459 |  | 0.008 (0.001, 0.016) | **0.032** |  | 0.005 (-0.002, 0.013) | 0.158 |  | 0.368 |  |
| **HPMM** | Ref |  | 0.006 (-0.002, 0.013) | 0.150 |  | 0.002 (-0.006, 0.009) | 0.634 |  | -0.007 (-0.015, 0.000) | 0.056 |  | **0.014** |  |
| Model was adjusted for age, sex, race, educational level, cotinine, smoking status, drinking status, Poverty Income Ratio (PIR), body mass index (BMI) and history of hypertension or diabetes. | | | | | | | | | | | | | |
| P for trend was calculated by treating quartile categories as a continuous variable. | | | | | | | | | | | | | |

| **Table S4.** Association between urinary creatinine-corrected mVOCs and serum LDH by exposure quartiles in fully adjusted linear regression models | | | | | | | | | | | | |
| --- | --- | --- | --- | --- | --- | --- | --- | --- | --- | --- | --- | --- |
| **Variable** | **Q1** |  | **Q2** | |  | **Q3** | |  | **Q4** |  |  | ***P* for trend** |
|  |  |  | **β (95% CI)** | ***P* value** |  | **β (95% CI)** | ***P* value** |  | **β (95% CI)** | ***P* value** |  |  |
| **2MHA** | Ref |  | -0.004 (-0.011, 0.004) | 0.319 |  | -0.011 (-0.018, -0.003) | 0.005 |  | -0.020 (-0.028, -0.013) | <0.001 |  | **<0.001** |
| **34MHA** | Ref |  | -0.018 (-0.025, -0.010) | <0.001 |  | -0.021 (-0.029, -0.014) | <0.001 |  | -0.035 (-0.043, -0.028) | <0.001 |  | **<0.001** |
| **AAMA** | Ref |  | 0.002 (-0.005, 0.010) | 0.569 |  | 0.003 (-0.004, 0.011) | 0.400 |  | -0.006 (-0.014, 0.001) | 0.097 |  | 0.971 |
| **AMCA** | Ref |  | -0.002 (-0.010, 0.006) | 0.614 |  | -0.006 (-0.013, 0.002) | 0.150 |  | -0.010 (-0.018, -0.002) | 0.011 |  | **0.023** |
| **ATCA** | Ref |  | -0.007 (-0.015, 0.001) | 0.068 |  | -0.016 (-0.024, -0.009) | <0.001 |  | -0.020 (-0.028, -0.013) | <0.001 |  | **<0.001** |
| **BMA** | Ref |  | -0.001 (-0.009, 0.006) | 0.732 |  | 0.006 (-0.001, 0.014) | 0.097 |  | 0.010 (0.002, 0.017) | 0.011 |  | 0.909 |
| **BPMA** | Ref |  | -0.002 (-0.009, 0.006) | 0.659 |  | -0.006 (-0.014, 0.002) | 0.116 |  | -0.008 (-0.015, -0.000) | 0.046 |  | 0.093 |
| **CEMA** | Ref |  | -0.001 (-0.008, 0.007) | 0.843 |  | 0.001 (-0.006, 0.009) | 0.730 |  | 0.006 (-0.002, 0.013) | 0.149 |  | 0.809 |
| **CYMA** | Ref |  | -0.003 (-0.010, 0.005) | 0.489 |  | -0.012 (-0.020, -0.004) | 0.002 |  | -0.021 (-0.028, -0.013) | <0.001 |  | **<0.001** |
| **DHBMA** | Ref |  | 0.011 (0.003, 0.019) | 0.005 |  | 0.021 (0.014, 0.029) | <0.001 |  | 0.031 (0.023, 0.039) | <0.001 |  | 0.095 |
| **HPM2** | Ref |  | 0.002 (-0.006, 0.009) | 0.640 |  | -0.008 (-0.015, -0.000) | 0.043 |  | -0.013 (-0.020, -0.005) | <0.001 |  | **0.036** |
| **HPMA** | Ref |  | 0.002 (-0.005, 0.010) | 0.521 |  | 0.001 (-0.007, 0.009) | 0.779 |  | -0.011 (-0.019, -0.004) | 0.004 |  | 0.278 |
| **MADA** | Ref |  | -0.004 (-0.012, 0.003) | 0.269 |  | -0.004 (-0.011, 0.004) | 0.338 |  | -0.008 (-0.016, -0.000) | 0.041 |  | 0.558 |
| **MHB3** | Ref |  | 0.006 (-0.002, 0.013) | 0.152 |  | 0.002 (-0.006, 0.010) | 0.621 |  | -0.010 (-0.018, -0.003) | 0.008 |  | **0.012** |
| **PHEM** | Ref |  | 0.012 (0.004, 0.019) | 0.002 |  | 0.012 (0.004, 0.020) | 0.002 |  | 0.017 (0.010, 0.025) | <0.001 |  | **<0.001** |
| **PHGA** | Ref |  | 0.005 (-0.002, 0.013) | 0.181 |  | 0.007 (-0.000, 0.015) | 0.058 |  | 0.010 (0.003, 0.018) | 0.008 |  | 0.371 |
| **HPMM** | Ref |  | 0.007 (-0.000, 0.015) | 0.067 |  | 0.006 (-0.002, 0.013) | 0.141 |  | -0.010 (-0.017, -0.002) | 0.013 |  | **0.015** |
| Model was adjusted for age, sex, race, educational level, cotinine, smoking status, drinking status, Poverty Income Ratio (PIR), Body Mass Index (BMI) and history of hypertension or diabetes. | | | | | | | | | | | | |
| P for trend was calculated by treating quartile categories as a continuous variable.   \| **Table S5.** RCS results for association between serum LDH and mVOCs \| \| \| \| \| \| \| \| \| --- \| --- \| --- \| --- \| --- \| --- \| --- \| --- \| \| **mVOCs** \| **UCr-uncorrected** \| \| \|  \| **UCr-corrected** \| \| \| \| **knots** \| ***P*-overall** \| ***P*-nonlinear** \|  \| **knots** \| ***P*-overall** \| ***P*-nonlinear** \| \| 2MHA \| 3 \| **0.002** \| **0.042** \|  \| 3 \| 0.087 \| 0.307 \| \| 34MHA \| 4 \| **<0.001** \| **0.003** \|  \| 4 \| **<0.001** \| **<0.001** \| \| AAMA \| 3 \| 0.712 \| 0.412 \|  \| 3 \| 0.712 \| 0.412 \| \| AMCA \| 3 \| **0.002** \| 0.074 \|  \| 3 \| **0.006** \| **0.035** \| \| ATCA \| 4 \| **<0.001** \| 0.080 \|  \| 4 \| **<0.001** \| **0.007** \| \| BMA \| 3 \| 0.238 \| 0.100 \|  \| 3 \| 0.094 \| 0.285 \| \| BPMA \| 3 \| **0.026** \| 0.207 \|  \| 3 \| 0.324 \| 0.861 \| \| CEMA \| 4 \| 0.057 \| 0.054 \|  \| 4 \| 0.187 \| 0.091 \| \| CYMA \| 3 \| **<0.001** \| **0.001** \|  \| 3 \| **<0.001** \| **0.002** \| \| DHBMA \| 3 \| **0.030** \| **0.049** \|  \| 3 \| **<0.001** \| 0.299 \| \| HPM2 \| 3 \| 0.109 \| 0.255 \|  \| 3 \| 0.738 \| 0.851 \| \| HPMA \| 4 \| **0.019** \| **0.008** \|  \| 4 \| **0.009** \| **0.005** \| \| MADA \| 3 \| 0.272 \| 0.263 \|  \| 3 \| 0.942 \| 0.990 \| \| MHB3 \| 3 \| **0.010** \| **0.036** \|  \| 3 \| 0.165 \| 0.139 \| \| PHEM \| 3 \| **<0.001** \| **<0.001** \|  \| 3 \| **0.000** \| 0.444 \| \| PHGA \| 3 \| 0.692 \| 0.544 \|  \| 3 \| 0.740 \| 0.662 \| \| HPMM \| 3 \| 0.051 \| 0.189 \|  \| 3 \| 0.061 \| **0.040** \| \| UCr-corrected models: lg(mVOCs/UCr) as exposure; UCr-uncorrected models: lg(mVOCs) as exposure. All models were additionally adjusted for age, sex, race, educational level, cotinine, smoking status, drinking status, Poverty Income Ratio (PIR), Body Mass Index (BMI, kg/m2) and history of hypertension or diabetes.  Knots were selected based on the smallest Akaike Information Criterion (AIC).  RCS: Restricted cubic splines; mVOCs: metabolites of volatile organic compounds; UCr: urinary creatinine. \| \| \| \| \| \| \| \| | | | | | | | | | | | | |

**Table S6.** Associations between urinary creatinine-corrected VOC metabolites and serum LDH levels in Generalized Linear Models

| **Variable** | **Model 1** | |  | **Model 2** | |  | **Model 3** | |
| --- | --- | --- | --- | --- | --- | --- | --- | --- |
|  | β (95% CI) | *P* value |  | β (95% CI) | *P* value |  | β (95% CI) | *P* value |
| **2MHA** | -0.013 (-0.019, -0.008) | **0.000** |  | -0.011 (-0.016, -0.006) | **0.000** |  | -0.006 (-0.012, 0.000) | 0.050 |
| **34MHA** | -0.023 (-0.028, -0.017) | **0.000** |  | -0.023 (-0.029, -0.018) | **0.000** |  | -0.022 (-0.029, -0.016) | **0.000** |
| **AAMA** | -0.008 (-0.016, 0.000) | 0.064 |  | -0.001 (-0.009, 0.006) | 0.733 |  | 0.009 (-0.000, 0.018) | 0.062 |
| **AMCA** | -0.011 (-0.018, -0.004) | **0.003** |  | -0.016 (-0.023, -0.009) | **0.000** |  | -0.011 (-0.020, -0.002) | **0.015** |
| **ATCA** | -0.017 (-0.023, -0.010) | **0.000** |  | -0.018 (-0.025, -0.011) | **0.000** |  | -0.016 (-0.022, -0.009) | **0.000** |
| **BMA** | 0.012 (0.005, 0.019) | **0.001** |  | 0.004 (-0.003, 0.011) | 0.254 |  | 0.007 (-0.000, 0.014) | 0.059 |
| **BPMA** | -0.007 (-0.011, -0.002) | **0.005** |  | -0.004 (-0.009, 0.001) | 0.081 |  | -0.004 (-0.008, 0.001) | 0.136 |
| **CEMA** | 0.009 (0.000, 0.017) | **0.038** |  | -0.005 (-0.013, 0.003) | 0.224 |  | 0.000 (-0.009, 0.009) | 0.971 |
| **CYMA** | -0.008 (-0.011, -0.005) | **0.000** |  | -0.007 (-0.010, -0.004) | **0.000** |  | -0.006 (-0.011, -0.001) | **0.019** |
| **DHBMA** | 0.059 (0.045, 0.073) | **0.000** |  | 0.039 (0.024, 0.054) | **0.000** |  | 0.051 (0.035, 0.066) | **0.000** |
| **HPM2** | -0.010 (-0.017, -0.004) | **0.003** |  | -0.009 (-0.015, -0.002) | **0.008** |  | -0.003 (-0.009, 0.004) | 0.449 |
| **HPMA** | -0.007 (-0.014, -0.000) | **0.044** |  | -0.006 (-0.012, 0.001) | 0.099 |  | 0.004 (-0.004, 0.012) | 0.330 |
| **MADA** | -0.008 (-0.018, 0.002) | 0.098 |  | -0.008 (-0.018, 0.001) | 0.081 |  | 0.002 (-0.009, 0.012) | 0.730 |
| **MHB3** | -0.009 (-0.015, -0.003) | **0.005** |  | -0.010 (-0.016, -0.005) | **0.000** |  | -0.005 (-0.013, 0.003) | 0.234 |
| **PHEM** | 0.017 (0.010, 0.025) | **0.000** |  | 0.015 (0.007, 0.022) | **0.000** |  | 0.023 (0.015, 0.031) | **0.000** |
| **PHGA** | 0.015 (0.004, 0.025) | **0.006** |  | 0.006 (-0.004, 0.016) | 0.267 |  | 0.018 (0.007, 0.029) | **0.002** |
| **HPMM** | -0.008 (-0.015, -0.001) | **0.023** |  | -0.012 (-0.018, -0.005) | **0.001** |  | -0.004 (-0.013, 0.005) | 0.366 |

Values are presented as β coefficients with 95% confidence intervals (CIs) and corresponding *P* values.

Model 1: Crude model (unadjusted).

Model 2: Adjusted for age, sex, race.

Model 3: Further adjusted for education level, Poverty Income Ratio (PIR), cotinine, smoking status, drinking status, Body Mass Index (BMI), and history of hypertension or diabetes.

| **Table S7.** Association between urinary mVOCs and serum LDH in fully adjusted linear regression models with FDR correction | | | |
| --- | --- | --- | --- |
| **Variable** | **β (95% CI)** | ***P* value** | **FDR-adjusted q value** |
| **2MHA** | -0.008 (-0.013, -0.002) | **0.005** | **0.014** |
| **34MHA** | -0.018 (-0.024, -0.013) | **<0.001** | **<0.001** |
| **AAMA** | 0.000 (-0.006, 0.007) | 0.952 | 0.952 |
| **AMCA** | -0.010 (-0.017, -0.004) | **0.002** | **0.007** |
| **ATCA** | -0.015 (-0.021, -0.009) | **<0.001** | **<0.001** |
| **BMA** | 0.001 (-0.005, 0.007) | 0.690 | 0.733 |
| **BPMA** | -0.005 (-0.010, -0.001) | **0.017** | **0.041** |
| **CEMA** | -0.004 (-0.011, 0.002) | 0.191 | 0.271 |
| **CYMA** | -0.007 (-0.012, -0.003) | **0.002** | **0.007** |
| **DHBMA** | 0.007 (-0.001, 0.015) | 0.076 | 0.118 |
| **HPM2** | -0.005 (-0.011, 0.001) | 0.076 | 0.118 |
| **HPMA** | -0.002 (-0.008, 0.005) | 0.590 | 0.669 |
| **MADA** | -0.004 (-0.011, 0.003) | 0.245 | 0.320 |
| **MHB3** | -0.007 (-0.014, -0.001) | **0.027** | **0.057** |
| **PHEM** | 0.021 (0.012, 0.030) | **<0.001** | **<0.001** |
| **PHGA** | 0.002 (-0.005, 0.009) | 0.544 | 0.661 |
| **HPMM** | -0.007 (-0.014, -0.000) | **0.040** | **0.076** |
| Values are presented as β coefficients with 95% confidence intervals (CIs) and corresponding P values. | | | |
| False discovery rate (FDR) correction was performed using the Benjamini–Hochberg procedure across the 17 metabolites in model 3. | | | |

P values were ranked in ascending order and adjusted using the formula q = P × m / i (where m = 17 and i represents the rank of each P value). Monotonicity of q values was ensured using the standard step-down procedure. Statistical significance after FDR correction was defined as q < 0.10.

**Fig. S1** Restricted cubic spline plots for the association between serum LDH and the first 9 mVOCs

**
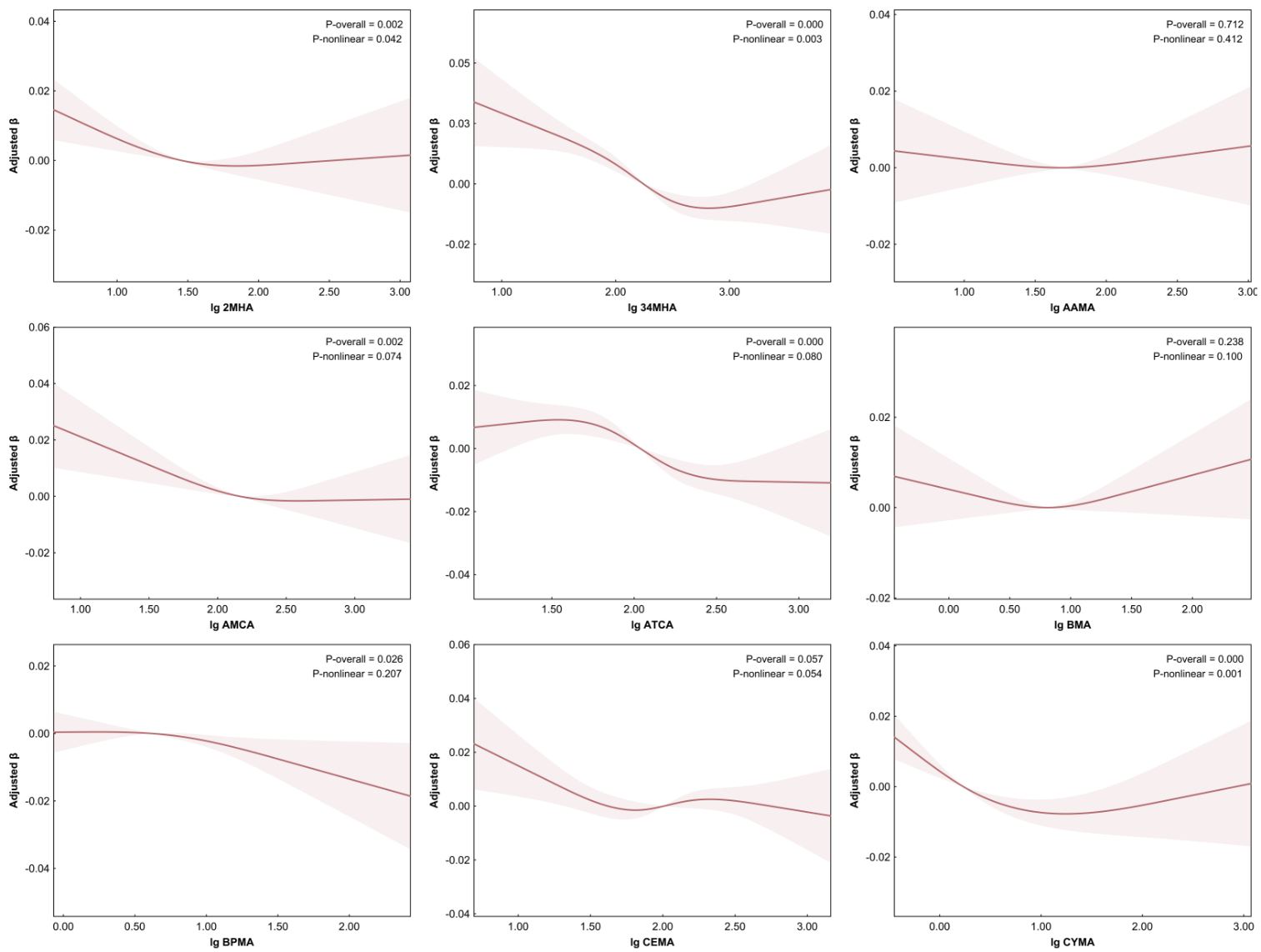
**

**Fig. S2**Restricted cubic spline plots for the association between serum LDH and the remaining 8 mVOCs


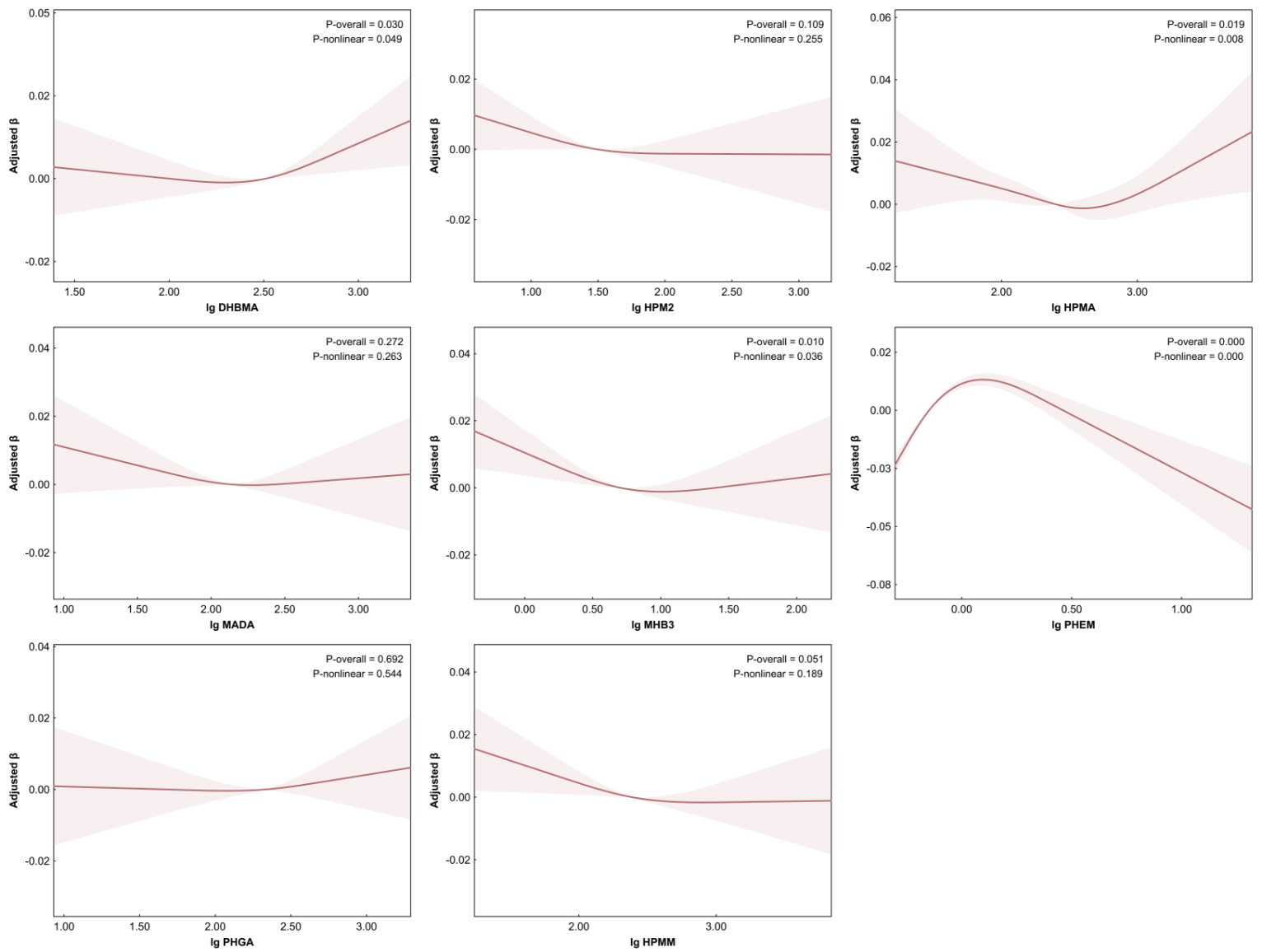


**Fig. S3** Restricted cubic spline plots for the association between creatinine-corrected mVOCs and serum LDH (first 9 mVOCs)


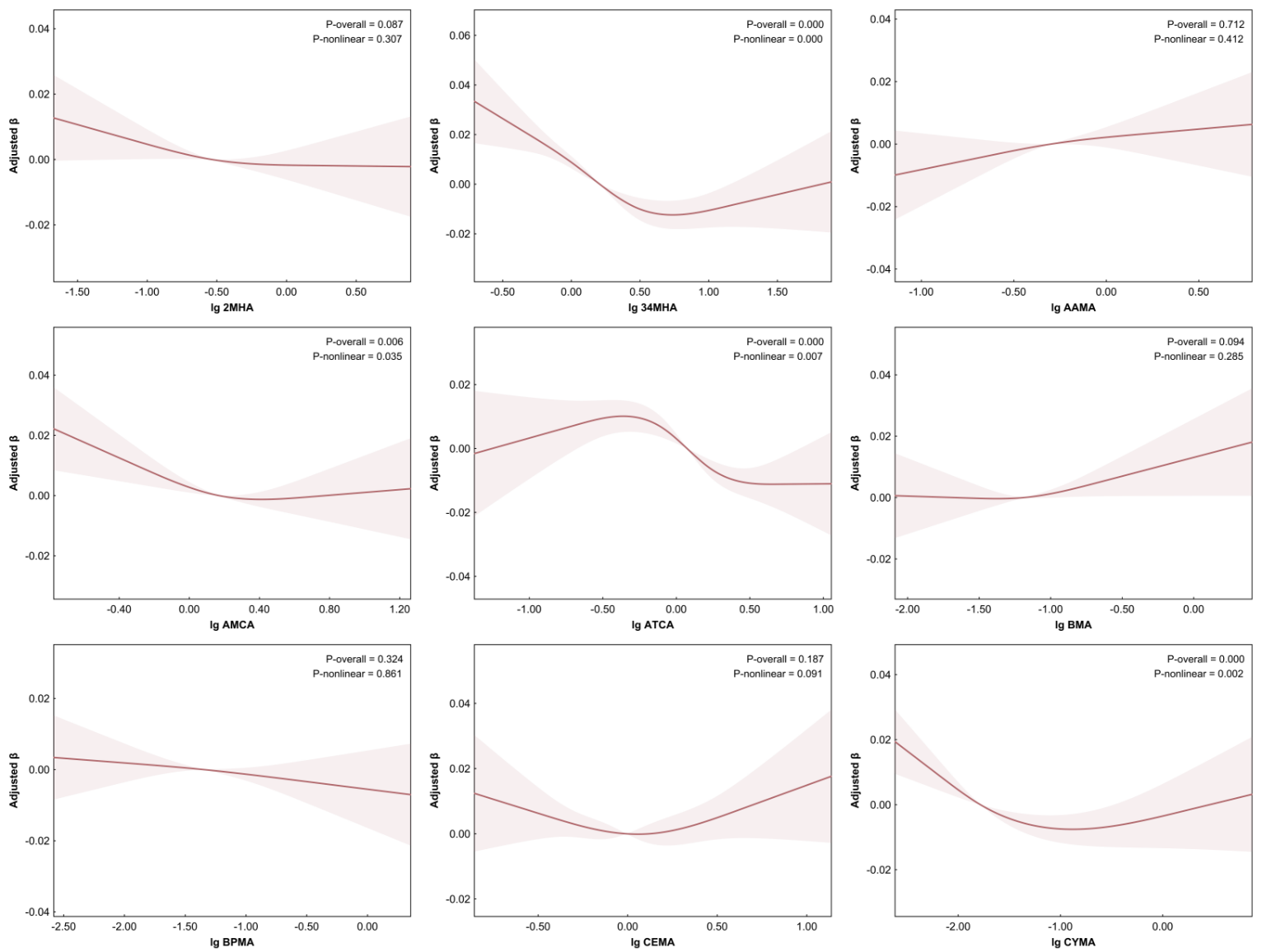


**Fig. S4** Restricted cubic spline plots for the association between creatinine-corrected mVOCs and serum LDH (remaining 8 mVOCs)


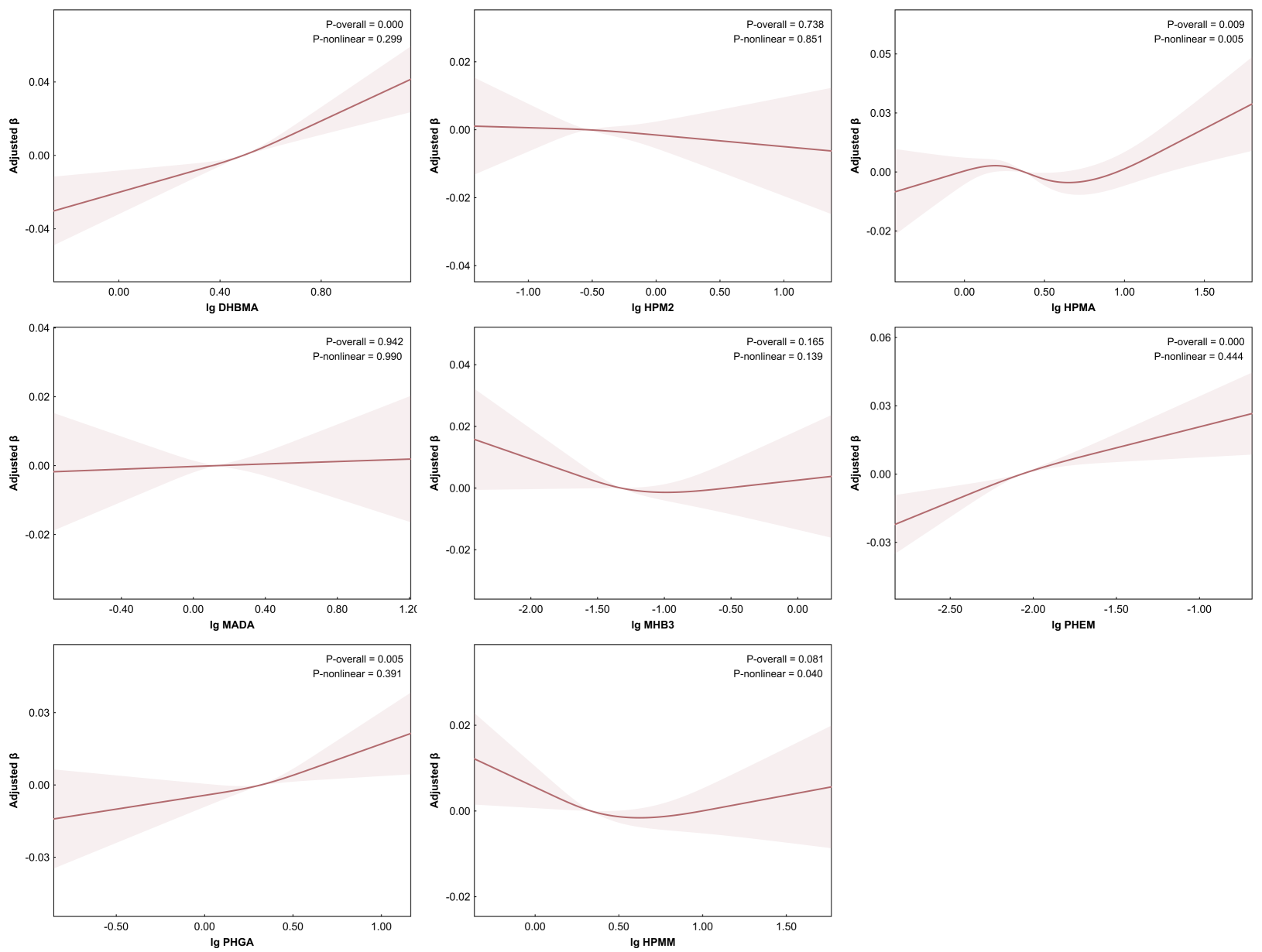


**Fig. S5** Weighted quantile sum regression results for the mixture effects of creatinine-corrected mVOCs on serum LDH


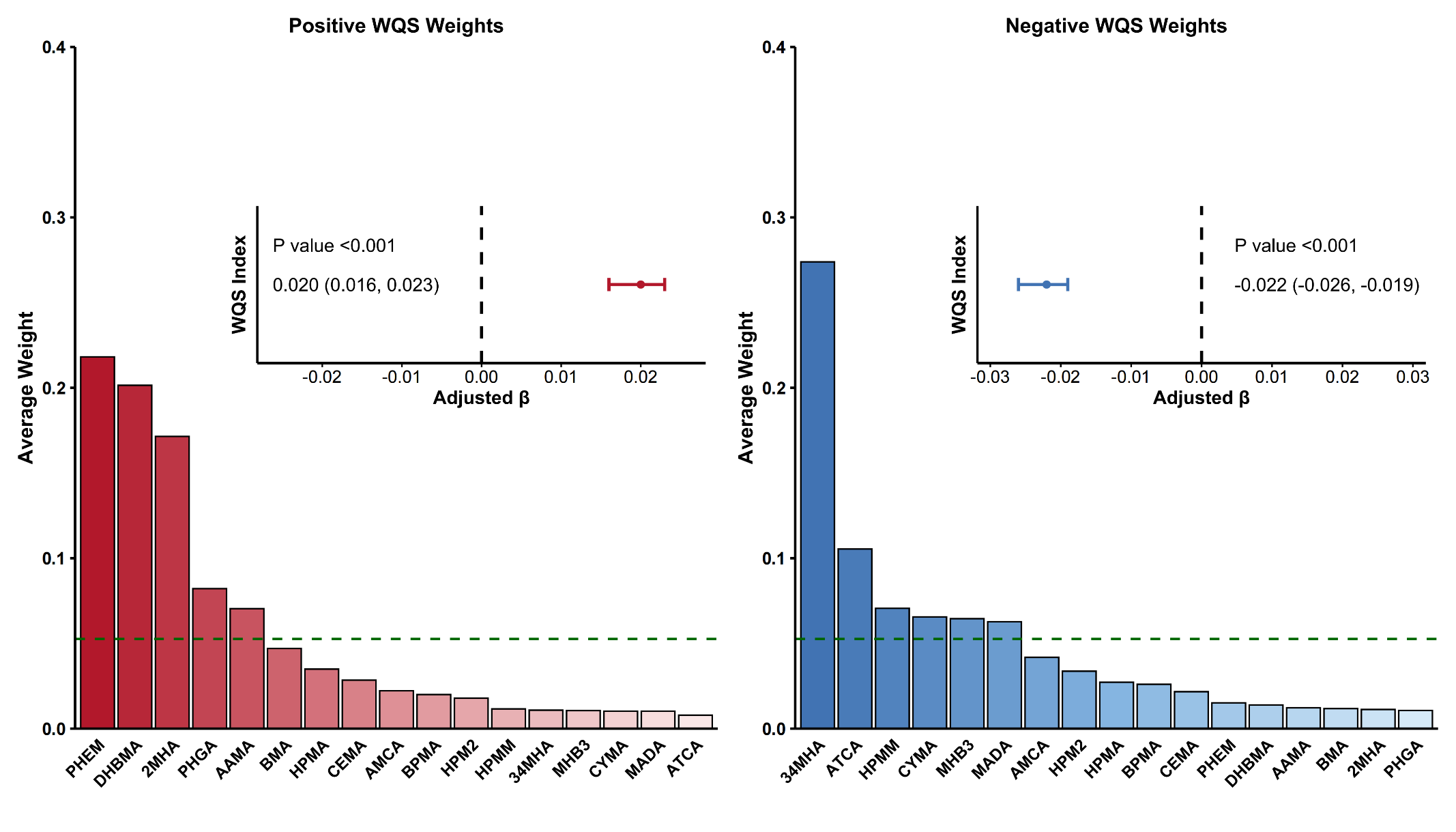


**Fig. S6** The quantile g-computation result for the mixture effects of mVOCs on serum LDH


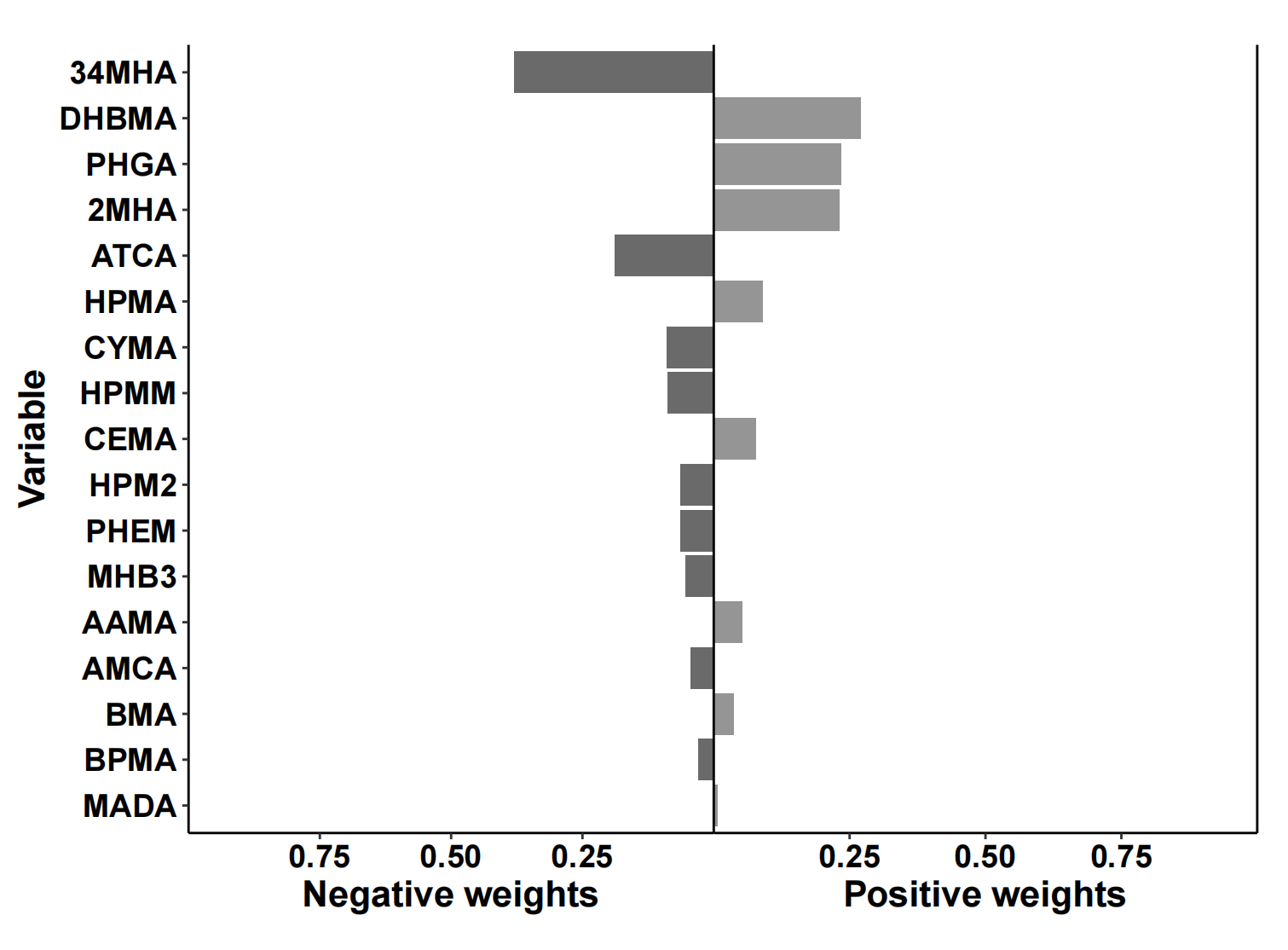

Supplement: Supplementary file 1 — Supplementary Material 1. [file 420_2026_2215_MOESM1_ESM.docx]
